# Metacognitive and Non-Metacognitive Processes in Arithmetic Performance: Can There Be More than One Meta-Level?

**DOI:** 10.3390/jintelligence10030053

**Published:** 2022-08-04

**Authors:** Csaba Csíkos

**Affiliations:** Institute of Education, University of Szeged, 6722 Szeged, Hungary; csikoscs@edpsy.u-szeged.hu

**Keywords:** metacognition, arithmetic, cognitive development, word problems

## Abstract

The nature of the development of arithmetic performance has long been intensively studied, and available scientific evidence can be evaluated and synthesized in light of Nelson and Narens’ model of metacognition. According to the Nelson–Narens model, human cognition can be split into two or more interrelated levels. Obviously, in the case of more than two levels, cognitive processes from at least one level can be described as both meta- and object-level processes. The question arises whether it is possible that the very same cognitive processes are both controlled and controlling. The feasibility of owning the same cognitive processes—which are considered the same from an external point of view of assessment—as both meta- and object-level processes within the same individual opens the possibility of investigating the transition from meta-level to object-level. Modeling cognitive development by means of a series of such transitions calls forth an understanding of possible developmental phases in a given domain of learning. The developmental phases of arithmetic performance are described as a series of transitions from arithmetical facts to strategies of arithmetic word problem solving. For school learning and instruction, the role of metacognitive scaffolding as a powerful educational approach is emphasized.

## 1. Introduction

Metacognition has long been recognized as a powerful term describing higher-level processes of intelligent human behavior. Starting from the theories and empirical results of metamemory, different metacognitive processes were described (Flavell 1979), and their theoretical and educational relevance proved to be highly visible.

Several investigations revealed straightforward relations between measures of metacognition and other domains, such as in Veenman et al.’s (1997) study, where intelligence, metacognitive skills, and academic achievement were involved in a university student sample. Nevertheless, other research found no direct or strong connections between intelligence and metacognition (Veenman and Elshout 1999). The Janus-faced connection between different aspects of metacognition and intelligence (Veenman et al. 2004) was longitudinally investigated by van der Stel and Veenman (2014), and one of their interesting findings suggested that metacognition has a relevant contribution to learning performance, and this contribution is partly independent of intelligence. Furthermore, it seems that the development of metacognition in young adolescents involves a shift from domain-specific to domain-general components, while the overall development seems to terminate. In these investigations, the measures of metacognition varied, and the interpretation of the relation between the different psychological constructs required careful consideration of the actual measures and devices used.

Nevertheless, the theoretical obscureness of the early models of metacognition led to a fragmentation of metacognition research (see e.g., Flavell 2004; Schneider 2008), while from the aspect of educational relevance, questions raised by practitioners about the practical relevance of metacognition in classroom settings have to some extent left unanswered. The ironic question of “Is it still worth knowing that 5 + 2 is 7, even if I could neither plan nor monitor the process of calculation?” could have been followed by more practical ones like “Is it worth consciously knowing the usual strategic steps of mathematical word problem solving even though the arithmetic skills do not allow the numerical data to be easily handled?” A kind of dichotomy seemed to be formed among in-service teachers about the relative weight and importance of metacognitive and non-metacognitive processes in the field of arithmetic. This ill-defined dichotomy may have led to confrontations between two camps (similarly to the struggle between the camps of the “mechanistic” and “realistic” approaches, see (Koninklijke Nederlandse Akademie van Wetenschappe 2009)), while theory development seemed not to make it obvious how both metacognitive and non-metacognitive processes play their important and dynamically changing role in the development of arithmetic skills. This essay attempts to reveal some relevant aspects of the balance between metacognitive and non-metacognitive processes in arithmetic performance, thus contributing to better education in the near future.

## 2. Theoretical Advancements and Empirical Results from the Last Decades

From the very early appearance of the term “metacognition” until the milestone article by Nelson (1996), most of the theoretical foci revolved around the possible dichotomies according to which the components of metacognition can be arranged and discussed.

### 2.1. Classical Taxonomies of Metacognition and the Nelson-Narens-Model

From early papers on metacognition that were primarily focusing on metamemory (Flavell 1979; Brown 1978), the need for categorizing different mental processes or structures that might be labeled as metacognitive has been put on researchers’ agenda. One of these attempts and efforts was the well-known dichotomy of declarative versus procedural, which seemed to be applicable to metacognitive processes as well. Kluwe (1987) created such a description of metacognitive knowledge components where declarative metaknowledge refers to beliefs or factual knowledge about one’s own knowledge or about knowledge in general. While procedural metaknowledge refers to controlling and regulation processes. A special kind of declarative meta-knowing is called meta-strategic knowledge by Kuhn (2000). The declarative–procedural dichotomy is similar to that coined by Paris and Winograd (1990) in which self-appraisal and self-management are differentiated.

Schneider (2008) summarized the available evidence on the different developmental pathways of declarative and procedural metacognition. Whereas declarative metacognition shows a straightforward developmental trajectory (due to the development of meta-strategic knowledge (see Kuhn 2000), components of procedural metacognition, i.e., the actual monitoring and control processes, do not show such clear-cut patterns.

Nelson’s (1996; see also Nelson and Narens 1994) seminal paper opened the perspective of talking about metacognition as one level of human cognition—while the other, the non-metacognitive level was labeled as object-level. The two types of information flow between the two levels were called controlling and monitoring. This article simplified the previous taxonomies of cognition and metacognition (see e.g., Darling et al. 1998) while opening new debates with three truly remarkable concerns: (1) The possibility and feasibility of more than one meta-level; (2) The issue of consciousness as necessary and/or sufficient condition for metacognition, and (3) The mind–brain correlates of metacognitive processes. As for the latter, the decisive role of the lateral prefrontal cortex has proven to be evidenced (Fleming and Dolan 2012). Moreover, Hikosaka and Isoda (2010) found that the lateral prefrontal cortex has a leading role in behavior switch. In Section 2.3, both the multi-level issue and the problem of consciousness at the meta-level will be briefly discussed.

### 2.2. System 1 and System 2

It is fairly obvious how relevant the connection between research using the terms System 1 and System 2 for our endeavor on the levels of human cognition can be. The terms originate in Stanovich and West’s (2000) oeuvre, and while the term System 1 depicts automatic, largely unconscious, and effortless processes, the term System 2 refers to more controlled, explicit, and demanding processes constrained by the limits of cognitive capacity. Thompson (2009) explicitly linked these two concepts to metacognition theory in a way that System 1 is basically a smooth-running system until a metacognitive process named “feeling of rightness” triggers System 2. The logic behind this assumption is of a brain-capacity-constraint nature, and the feeling of rightness, i.e., the feeling that things are on their right way, assumes metacognitive monitoring processes. Although a strong critique of each two-level and dichotomized model was expressed by Keren and Schul (2009), both the Nelson–Narens model of metacognition and the System 1 and 2 duet found their fruitful educational applications in basic skills instruction, for which we also intend to provide further insights.

### 2.3. More than Two Systems in Human Cognition

The possibility of more than one meta-level was explicitly described by Nelson and Narens (1994). There is a starting level that acts as an object-level only (L_0_) and L_1_, L_2_, L_N_ would ensign the several (N) possible meta-levels built on it. Independently of the actual number of Ls, it is their relative position that counts, e.g., L_j−1_ is the object-level of L_j_. Theoretically, one can describe as many meta-levels as desired. As Nelson (1996, p. 105) claimed, “Any lower-level cognition can itself be the subject of a higher-level cognition”, and lower-level and higher-level cognition can occur simultaneously.

However, as Levinsson (2008) argued, the problem with the endless number of possible levels is that humans seem to have limitations or endpoints in their reasoning. Having seen the grotesque wording by James Joyce, people would usually get along with only two or three levels.

What, reduced to their simplest reciprocal form, were Bloom’s

thoughts about Stephen’s thoughts about Bloom and about Stephen’s

thoughts about Bloom’s thoughts about Stephen?

—James Joyce, *Ulysses*

As Schatteles (2014) analyzed, unraveling the meaning of this awkwardly complex sentence may require a series of step-by-step sentences which gradually unfold the meaning of the original sentence.

One further aspect that was formulated as a debate in Nelson’s (1996) article is the issue of consciousness in (relation to) metacognition. The control processes at the meta-level do not necessarily involve conscious processes (Levinsson 2008; Veenman et al. 2006; Timmermans et al. 2012). Nonetheless, the possible existence of non-conscious meta-levels would strengthen the assumption that the existence of more than one meta-level is feasible.

A similar description of more than two possible levels of cognition appeared in the discourse community of consciousness studies. Schooler (2002) suggested three levels of mental processes to be distinguished: unconscious, conscious, and meta-conscious processes. The latter involves a kind of re-representation of conscious processes. Since the relation between metacognition and consciousness is still an ongoing debate (Rosenthal 2012), Schooler’s model is considered as opening the door to multi-level and multi-dimensional models.

The questions may arise whether the levels (independently of their numbers) are different mental representations within the individual and/or whether the levels can be objectively described. This dilemma is similar to the one explicated by Rosenthal (1998) about first-person versus third-person consciousness. From an educational point of, we would go in the direction of a third-person, objective description of the possible levels of cognition, nonetheless allowing the assumption that object-level processes for one person can be meta-level processes for another and vice versa.

With the involvement of the conscious versus non-conscious debate, an extension of the original one-dimensional, two-level model of metacognition became viable. Fleming et al. (2012) extended the Nelson–Narens model of metacognition in a way that the object- versus meta-level distinction is one out of three dimensions, and consciousness and the observable behavior form two additional dimensions. In this three-dimensional model, metacognition may have several facets, and empirical investigations are invited to test whether the three dimensions are independent of each other.

## 3. Two Educationally Relevant Dilemmas Concerning the Metacognitive and Non-Metacognitive Processes of Arithmetic Performance

With the introduction of the term “metacognition”, an immediate and necessary dichotomy of metacognitive versus non-metacognitive components appeared. With more or less precision, researchers (e.g., Zohar and Ben David 2009; Tanner 2012) collected and described the psychological processes that may (or even should) be labeled as metacognitive. In the field of arithmetic performance, researchers usually call these metacognitive processes strategies (Campbell and Xue 2001; Park and Brannon 2014; Cragg et al. 2017), or sometimes other labels are used, such as conceptual understanding (Gilmore and Bryant 2008; Gilmore and Papadatou-Pastou 2009). Two serious questions arose that certainly have educational relevance, and there is a scarcity of empirical studies in order to find definite answers. The first question concerned whether the very same performance from two individuals may be due to very different underlying processes, i.e., solving the same problem with or without metacognitive processes such as planning or monitoring. The second question concerned the measurable difference between the metacognitive processes currently used and the metacognitive processes potentially available to the same person.

### 3.1. The Balance between Metacognitive and Non-Metacognitive Processes of Arithmetic Performance

The same level of performance may be reached in very different ways. For example, in the field of reading, the compensatory-encoding model (Walczyk 1995) claims that compensatory mechanisms—which are metacognitive skills such as slowing reading rate and re-reading parts of the text—enable the reader to compensate for subcomponent inefficiencies (Afflerbach et al. 2008). Although the terminology may vary, a kind of balance between metacognitive and non-metacognitive components can be described in different models of reading comprehension. In the field of arithmetic skills, fairly different calculation strategies have been identified even among kindergarten children (Zur and Gelman 2004) resulting in the same or similar performance. In a study by Csíkos (2016), elementary students’ three-digit mental calculation performance proved to be of the same level in two schools; however, their reported strategy use differed quite remarkably.

As a corollary of the question of individual differences in the balance between metacognitive and non-metacognitive processes, there emerged the idea of possible developmental transformations in this balance. Leahey and Harris (1993) questioned the earlier theories of a more or less straightforward developmental trend where metacognition plays its increasingly important role in human capabilities. “What happens during the transition from conscious thought to intuition is contentious” (pp. 284–85). They had developed some arguments on the apparent contradiction between the increasing availability of mental resources and the limited or seemingly unchanging capacity of attention and memory.

From an educational point of view, the essence of the debate is summarized as “what had formerly required conscious thought becomes intuitive, and an important question concerns what happened to the rules followed consciously by the novice...” (Leahey and Harris 1993, p. 284). This paradigm can be nicely illustrated with everyday situations. For instance, the novice driver may learn and be potentially aware of the MSM rule (mirror, signal, maneuver), and while later they can follow this sequence very well and automatically, it would be strange to still quote or murmur the acronym once learnt. However, in difficult situations or while being exhausted behind the wheel, the expert learners may still be capable of recalling and following this sequence, since it has remained potentially available to them. From a distant observer’s point of view, when a car starts smoothly from a parking lot, it is not easy to judge whether the driver needed some conscious (and metacognitive) support or not.

If the same level of performance can be reached with or without metacognitive processes as well, could it be possible that performance attained without metacognitive processes indicates a kind of new developmental level? This debate seems to be controversial since intuitively the appearance of metacognitive processes may indicate a higher developmental level. This thought has already been addressed in the literature, e.g., Sternberg (1998, p. 129) claims that, “when functioning is automatic, metacognitive activity can actually hamper functioning”. A precursor to this idea was expressed in his seminal Triarchic mind book (Sternberg 1985, p. 62): “It would be very difficult to speak intelligently if we had to consciously struggle to come up with every word.” Obviously, fluent speakers, professional drivers, and musicians can fluently speak, drive, or play without time-consuming decision and monitoring processes. As Polanyi (1958) reasoned, the pianist would surely make a mistake if he or she tried to be aware of all aspects of the actual piano playing.

### 3.2. Difference between Actual and Potential Metacognitive Processes of Arithmetic Performance

Now we address the problem of making distinction between the currently or actually used metacognitive processes, and the potentially available metacognitive processes. The dilemma can be articulated as follows. By all means, possessing a repertory of strategies can support human problem solving in many domains. However, the individual preferences and the task characteristics may hinder or facilitate the use of those strategies. Do we want to observe or improve a strategy as a function of task and individual characteristics, or are we just content to know about the potential availability of that strategy? A similar idea was raised in intelligence research by Danthiir et al. (2005) when addressing the issue of measuring mental speed. Investigations usually attempt to measure mental speed as a kind of typical behavior (as opposed to the potentially available maximum behavior). The analog version of maximum behavior may be the repertory of potentially available metacognitive resources, and the actually used metacognitive processes may be the analogue of the typical behavior. Sun et al. (2021, p. 12) have already addressed the problem of whether researchers measure the actual or the potential level of metacognition in the field of foreign language learning: “we measured EFL learners’ metacognitive experiences after they finished a writing task, but students’ metacognitive experiences are dynamic during the writing process”. As Pintrich et al. (2000) emphasized, construct validity of the measures of metacognition depends not only on the empirical evidence a test may provide but on the potential consequences of the interpretation of the test data.

In the field of arithmetic performance, for the average adult, simply mentioning the addition 5 + 2 will yield an answer (pronounced out loud or not) without actually asking them for the results. They probably possess the result as a number fact that can be easily retrieved. Therefore, we can hardly imagine that adults can honestly report on their mental calculation strategies while attaining the result of such an easy task. Yet we do not think that the lack of strategy use made them struggle, and of course, we do not claim that kindergarten children solving the very same addition task by using their fingers and counting aloud from five to seven are more proficient. However, they did exhibit a rich repertory of strategic processes in this case. “Metacognition…is not likely to be… a separate ability that once acquired can then be universally applied to different types of mental activity. Instead, metacognition is more likely to develop in tandem with the more basic cognitive abilities and conceptual understanding in each domain” (Estes 1998, p. 1346).

In Nakakoji and Wilson’s (2020, p. 12) research among university students and academics, many surprising findings were yielded: “Also unexpectedly, neither students nor academics mentioned metacognition in the interviews.” However, for the researchers it was evident that metacognitive strategies were employed. According to Nakakoji and Wilson’s conclusion, the participants may not have been aware of their strategy use. In EFL-learning situations, similar lack of metacognitive strategies was detected by Zhang et al. (2021). This may be due to the inappropriateness of the think-aloud protocol or the questionnaire they applied, or there were really no strategic processes potentially reportable. Similarly, a review of studies with gifted children (Alexander et al. 1995) concluded that albeit declarative metacognition seems to develop with age, the development of procedural components of metacognition shows intricate trend lines.

Consequently, several kinds of measures of metacognition can be defined according to individual characteristics (like age) and task characteristics (like the number of digits of the addends). Furthermore, there is another crucial factor influencing the actual strategy use: the context in which the individual encounters the task—whether it is a stressful situation or just a mental calculation for fun. All these factors influence whether metacognitive processes (if available at all) may or should play their role in arithmetic or other kinds of performance. These factors will be again discussed in Section 4.1 and Section 4.2 as the three main aspects of adaptive expertise. Furthermore, the several possible types of measures of metacognition can be arranged whether they intend to measure the actual or the potential availability of metacognitive processes, or whether they assess (verbally) reportable metacognitive components or those that can be observed by an external viewpoint. The currently available measures of metacognition address one or more metacognitive components, but none of them can be considered as the ultimate or complete measure of metacognition.

Some widely accepted and used measures of metacognition like MAI (Gregory Schraw and Dennison 1994), MARSI (Mokhtari and Reichard 2002), and the several questionnaires of epistemological beliefs (e.g., Schraw et al. 2002) seem to measure a kind of static, potentially available repertory of metacognitive components. Other measures could grab the currently working components of metacognition. Of course, the actually used components cannot really be measured by questionnaires, but observations, think-aloud protocols, and eye-tracking (Paulus et al. 2013) may reveal them. On the other hand, as Kavousi et al. (2020, p. 711) claim, “metacognition cannot be measured directly through observation”. Convergent findings from both online and offline measures can be used to detect both the actually used and the potentially available metacognitive components!

Azevedo et al.’s (2010) study represents six potential trends in the timeline changes of self-regulated learning activities. Albeit the authors acknowledge that it is exceptionally difficult to capture self-regulated learning activities in a hypermedia environment, they dare judge the potential frequency of possible trend lines. One of these trend lines is of inverted U-shape (the authors judge its observable empirical frequency low) when an initially low state of self-regulated activities increases and then returns to the original level. Independently of the age and task characteristics, this inverted U-shaped line may demonstrate how strategy use occurs in a wide range of novel learning situations, including for instance arithmetic calculations.

## 4. Educationally Relevant Answers in the Field of Arithmetic Performance

### 4.1. (At Least) Three Levels of Components in Arithmetic Performance

**Figure 1:**
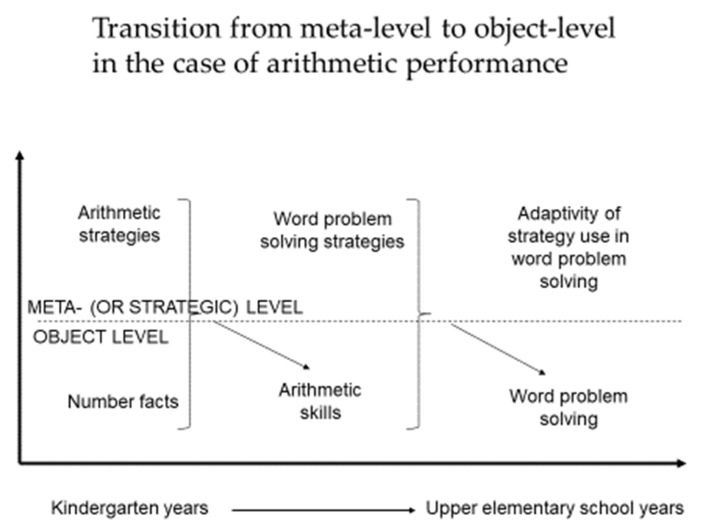
A tentative model of the development of arithmetic performance in the light of the Nelson–Narens model of metacognition.

As visualized in Figure 1, we propose a model consisting of three developmental phases with two levels of mental processes. This way we intend to keep and apply the traditional Nelsonian two-level model of metacognition while putting forward a developmental model of arithmetic performance that involves and integrates the currently available empirical findings. We may claim that this model is plausible or at least possible; however, it does not necessarily follow from the already existing research results. However, we do think that this model may be informative and illuminating in the context of teaching and learning mathematics and other school-related domains.

According to the model, in the first phase of development, it is the number facts (e.g., results of simple one-digit additions) that comprise the object level, and the fledgling arithmetic strategies are on the meta-level of processing. The object level is characterized by automatic, effortless functioning. Logan (1988) claims that when describing automatic processes, it is wise not to refer to them as counterparts to non-automatic processes (such as unconscious vs conscious, effortless vs effortful, etc.), but it is worth providing quantitative properties of automatic functioning in terms of, for instance, reaction time or flawlessness (e.g., error-free execution of operations).

Hudson et al. (2018) and Zur and Gelman (2004) examined kindergarten children’s addition skills with special emphasis on their strategy use. In this first phase of arithmetic performance development, the automatization of simple number facts and the diversification of a repertory of counting strategies are observable. Fact retrieval is associated with different brain regions than calculation strategies (Zarnhofer et al. 2013). Furthermore, fact retrieval requires stable verbal representations as well (Berteletti et al. 2014), and in the brains of children with mathematical disabilities, the activity of the verbal regions was found not to be reliable.

During the first years of elementary schooling, students’ arithmetic skill develops, and this development is the result of a balanced use of metacognitive and non-metacognitive processes. (Here the term metacognitive and non-metacognitive is used in the sense of their position in the first developmental phase.) In the second phase, the more or less automatized arithmetic skill is put to another challenge; it must be used not only in pure numeric tasks but in word problems as well. Even the very first, usually routine-like word problems enforce students to learn new strategies, which are about the expected use of their arithmetic skills in a new context. As Csíkos and Szitányi (2020) pointed out, several textbooks provide linearly sequential steps or algorithms on how to solve routine word problems.

During several months or years, and due to the obedient adherence to the rules (both classroom rituals and the required word problem solving steps, see this idea from Brousseau in D’Amore and Martini 1999), the majority of students become proficient in (routine) word problem solving (Lee et al. 2011). However, as seen from many empirical studies, this new phase of automatization causes serious problems when realistic word problems are administered (see, e.g., Verschaffel et al. 2010).

The next developmental phase can, therefore, be a new strategy learning phase when students learn to choose the word problem strategy adaptively. Adaptation in this phase will primarily take the characteristics of the tasks into account, but individual preferences (e.g., visualizers versus verbalizers) and contextual variables (high-stake testing versus classroom practice) also play their role in determining what counts as an adaptive strategy. In order to provide the best actual performance, the balance of metacognitive and non-metacognitive components can vary according to several factors. These factors have been enlisted (and empirically validated) in research on adaptive expertise in elementary mathematics (Verschaffel et al. 2007, 2009) and were proposed as general factors of adaptive intelligence by Sternberg (2021).

The very same person may be characterized by different balances of metacognitive and non-metacognitive processes in different tasks and in different contexts. A more challenging task may require a higher-level (or in certain circumstances: lower level) involvement of metacognitive processes, and an exam-like stressful situation may further influence the involvement of metacognitive processes even for the same person and the very same task. This kind of flexibility requires “adaptive expertise”—a term coined by Hatano and Inagaki (1986) as an opposition to “routine expertise”, which refers to quick and accurate functioning without much understanding (De Corte 2007).

Finally, when talking about developmental phases as depicted in Figure 1, we do not talk about individuals’ developmental phases, but these are developmental phases of arithmetic performance. Therefore, we cannot state that a student or an adult is at a certain developmental phase, since depending on individual characteristics, task, and context variables, the very same person may exhibit different performances. Individual characteristics include what Sternberg et al. (2021) call meta-intelligence, i.e., the psychological construct responsible for the allocation of mental resources and controlling that allocation. Differently from the concept of metacognition, meta-intelligence comprises constructs that are rather conative such as creativity and wisdom.

Since we do not talk about individual developmental phases but about the developmental phases of observable performance, the question arises whether adults’ learning may be characterized by the same balances of metacognitive and non-metacognitive components of arithmetic performance. Zamarian et al. (2018) revealed that for enhancing arithmetic performance, adults can also profit from repetition-based training but to a lesser extent than young people. The difference may be attributed to children’s limited capacity of executive function in the mind and different functioning of the brain. Adults’ brain activity showed a more distributed pattern than those of younger individuals’ brain where the right parietal brain structures proved to be associated with arithmetic performance. In their most recent investigation, Grabner et al. (2022) revealed that adults primarily solve single-digit addition tasks by simple number fact retrieval, instead of applying (however quick or unconscious) counting procedures.

### 4.2. Transition between Phases of Arithmetic Performance

Figure 1 suggests transition processes between the developmental phases of arithmetic performance during the elementary school years. Nevertheless, the arrows depicting transitions do not intend to indicate causal relations in any directions. It is the task of future longitudinal research to reveal the nature of developmental transitions, and how education can help in triggering development. As for the first arrow illustrating that arithmetic skills may become object-level components, we would like to emphasize that it is neither the number facts nor just the fledgling arithmetic strategies that become automatic, but arithmetic skill comprises a mixture of metacognitive and non-metacognitive processes. It means that learners may possess very different mastery levels of arithmetic skills in the second phase when those skills become object-level components for word problems.

The very different balances or mixtures of metacognitive and non-metacognitive processes in children’s arithmetic skills have been addressed in several previous studies. According to Flavell (2004), this age group is going through striking improvement in different areas of metacognition. How arithmetic performance may develop within the first phase was summarized and empirically evidenced by Lemaire and Siegler (1995) who revealed four aspects of strategy change in elementary school students’ multiplication performance, “introduction of new strategies, shifts toward greater use of the more efficient existing strategies, improved execution of the strategies, and more adaptive choices among the strategies” (p. 96).

Carpenter and Moser (1984) revealed that a variety of counting strategies appear even before receiving formal instruction on them. Their study was restricted to strategy use, and they claim their model does not take account of children’s knowledge of number facts. Moreover, Zur and Gelman (2004) and Pappas et al. (2003) provided evidence about the existence of rudimentary metacognitive arithmetic strategies. Siegler (1987) found among young children (kindergarten and 1st and 2nd grade) that in very simple addition tasks like 5 + 2 or 4 + 1, retrieval produced faster and equally appropriate answers than using one of the simple strategies like counting on from the larger number. In a study comparing disabled and non-disabled children, Ostad (1997) found that disabled children used more simple strategies and there was not much diversity in their strategy use. Their most common strategies were counting on fingers or counting audibly. Interestingly, they rarely used the strategy of immediate retrieval from long-term memory. In contrast, their mathematically non-disabled peers could more and more frequently use the retrieval strategies, allowing for the implication that possessing and recalling number facts may indicate a higher-level of arithmetic performance in the case of very simple arithmetic tasks. Furthermore, Canobi (2004) found that finger-based counting strategies almost disappear during the first years of schooling.

In sum, meta-level processes of arithmetic performance become visible and develop in tandem with object-level components, comprising a more or less automatized skill usually labeled as arithmetic skill(s). Whether an actual process is of meta-level or object-level depends on individual and task characteristics, and on contextual variables. When word problems are introduced (often as early as the very first grade of formal schooling), some children already possess smoothly functioning arithmetic skills (as a system of metacognitive and non-metacognitive processes), while others rely more on the fledgling arithmetic strategies.

Azevedo et al.’s (2010) proposed inverted U-shaped trend line about the increase and then decrease of self-regulation processes has been empirically evidenced by Bellon et al. (2019). In the first developmental phase of arithmetic performance, meta-level processes first have an increasingly important role and then retreat into the background, giving a primary role to number facts and retrieval processes. Grabner and De Smedt (2012, p. 10) nicely described this shift: “The current findings show that the well-known behavioral shift from effortful procedural strategies to fact retrieval strategies as a function of training is also reflected in specific changes in brain activity”.

The observable change in the brain activity, i.e., changes in the mind–brain correlates, makes the transition from the first to the second level especially interesting and special. Spanoudis and Demetriou (2020) identified domain-specific brain networks that are specialized regions associated with various mental processes. The development of brain networks for domain-specific mental processes (such as quantitative reasoning, which is the fundamental basis for arithmetic performance) takes place early. The basic range for the development of arithmetic fundamentals is between 7 and 11 years (Peters and De Smedt 2018), which is the usual period of elementary schooling.

It may sound reassuring and confirming how research results on mind development and its brain correlates (Spanoudis and Demetriou 2020) can unite the development of arithmetic skills and their accompanying metacognitive processes. Arithmetic operations belong to the realm of domain-specific skills, and due to the development of the mental representations and their corresponding brain regions, by the age of six, most children are ready for acquiring new representations. This does not mean that they still have to wait for about five years until reaching the so-called “cognizance” mental representation system where self-awareness and meta-representations provide a new level of flexibility and adaptivity. According to Spanoudis and Demetriou, even at the second developmental stage, there are mental processes that provide awareness about the actual, overwhelmingly domain-specific representations.

As for the transition from the second to the third phase, a plethora of empirical studies are available from the last four decades (e.g., De Corte and Verschaffel 1987; Rittle-Johnson and Alibali 1999; Selter 2009; Prediger and Krägeloh 2015). During their first years of schooling, students learn how to solve arithmetic word problems. The solution process itself becomes more and more automated. This automatization can be observed and documented in children even without having smooth, high-level functioning of arithmetic skills. However, having become automated word problem solving strategies may be object-level processes when students encounter different genres of word problems. It seems that at least two classes of elementary word problems should be distinguished in this second phase when talking about the role of metacognitive processes.

Routine word problems can be solved by a straightforward application of a “superficial” task solving strategy. These tasks represent a task category that requires students to apply their arithmetic skills in the context of applying a uniform solution strategy: collect the numerical data, find one (or more) appropriate arithmetic operations, execute those operations, and give a numerical answer. On the other hand, the most important feature of the realistic word problems is that they require at least one extra step in the solution process, i.e., the appropriate mental representation of the phenomena written in the text of the word problems, since the straightforward application of the above-mentioned superficial solution strategy would result in failure. The difference between the word problem solution based on appropriate mental representations and the solution based on the superficial “direct translation” strategy was documented by Hegarty et al. (1995).

Due to the variability of the involvement of metacognitive processes in adaptive expertise, it is not surprising that when Jacobse and Harskamp (2012) administered word problems to grade 5 students together with the metacognitive self-regulation subscale of the MSLQ questionnaire (Pintrich and De Groot 1990), an intricate connection between the two constructs was found. Students in their 5th grade of formal schooling can belong to very different developmental phases of arithmetic performance; in other words, some of them need strategic planning and monitoring while executing simple arithmetic operations, others feel themselves confident in implementing the required steps of routine word problem solving, while others may hesitate on whether realistic constraint should be taken into account or should they just follow the usual superficial solution strategy. The first and third groups may be found as active strategy users, but the second group may not show much conscious effort in word problem solving.

In Van der Stel et al.’s (2010) study, secondary school students solved word problems. These tasks may have required several consequent solution steps to take; therefore, students were trained in brief sessions on how to solve these kinds of problems. The deliberate use of different solution strategies inherently requires metacognitive skills like planning and monitoring; thus, their results are really about the role of metacognition in word problem solving. The connection between metacognitive skills and mathematics performance was stronger in the 14–15 than in the 13–14-year-old group. Based on large sample empirical results, Träff (2013) suggested a developmental model that reinforces Fuchs et al.’s (2010) results in that working memory constraints play a significant role in the development of arithmetic performance. Additionally, he joined the constructs of fact retrieval, multi-digit calculation skills, and word problem solving within one research design.

Similar tentative models may be sketched for reading and writing performance as well. For reading, the respective processes may range from phonological awareness to adaptive reading strategy use (Paris and Hamilton 2009), while for writing, the processes may range from the basic geometric elements of handwriting to composing a responsive essay. Hopefully, empirical evidence will be increasingly available about the existence of the proposed object- and meta-level developmental stages and about the process of transition. In reading, Wagoner (1983) used the term comprehension monitoring as covering several conscious, metacognitive processes (involving fix-up strategies as well) related to the comprehension of an actual text. As a matter of a debate, she contrasted two opposing branches of empirical results: whether both good and struggling readers show comprehension monitoring behavior or is it mainly reserved for the good readers? As Desoete (2009) revealed, adults with comorbid difficulties in both mathematics and reading tended to use their cognitive resources for consciously monitoring their activities that should have otherwise been more automated in people without disabilities.

Paralleling the object-level and meta-level components of mathematics set forth in Figure 1, and the possibly analogous components of reading, the following tentative comparisons can be given. Grapheme-to-phoneme reading is the starting object-level (see Steffler et al. 1998), while phonological and morphological awareness are on the starting meta-level (Tánczikné Varga et al. 2020). Decoding skills comprise the second object-level and reading strategies (in the sense defined by Almasi and Fullerton 2012; Afflerbach et al. 2008). Finally, adaptive strategy use may represent the highest developmental stage (He 2008). Although the empirical investigation of the mechanism of transitions between different developmental stages of arithmetic performance is a great challenge in itself, the parallel study of arithmetic and reading may bring further evidence on the effectiveness of the Nelsonian model of metacognition, at least in terms of educational aspects.

## 5. Corollaries

### 5.1. Observing and Measuring Metacognition in Arithmetic Performance

In general, the assessment of metacognition suffered from several weaknesses and has walked many paths. As noted by Baker and Cerro (2000), it is difficult to develop measures of metacognition that fulfill the traditional criteria of reliability and validity. The most frequently used questionnaires have serious limitations. First, they can only be used from the second grade of schooling at the earliest. One of the widely accepted measures of young children’s metacognition, the junior MAI (Metacognitive Awareness Inventory, Sperling et al. 2002) is suitable from grade 3. Second, the questionnaire method suffers from possibly false memory reconstruction and the prompting effect may be detected (see Veenman 2011). The prompting effect refers to the possibility that students often feel they have to come up with an answer, and what is more, they should come up with the “right” answer. Consequently, having labeled the questionnaires as an offline technique, several different online techniques appeared from think-aloud protocols through eye-tracking to the observation of behavior.

Online and offline measures weakly correlate with each other (Jacobse and Harskamp 2012), suggesting that the lack of connection may be due not only to the different platforms or means of assessment but also to the independency of two constructs measured separately by the two kinds of devices. Since think-aloud protocols comprise a branch of interviews, the attempt to construct interview methods that may yield the interviewee’s subjective experiences with great precision is definitely welcome (Petitmengin 2006). Think-aloud protocols grab the very moments of conscious metacognitive experiences, since they can be used to detect sequential processes (Jausovec 1994), logfile analysis in a computerized environment may provide additional information about metacognitive processes (Veenman et al. 2014). Furthermore, eye-movement research may help in detecting conscious, meta-level planning and monitoring activities during word problem solving (Csíkos and Steklács 2015). Besides, we would like to emphasize the need for more longitudinal studies, which would allow us to test the hypothetical developmental changes depicted on Figure 1.

For teachers who would like to collect valid information about their students’ metacognitive processes, open classroom discourse is a powerful method. While for some students, any question that goes beyond the task “What is 5 + 8?” and wonders “How could you calculate 5 + 8” (see Ginsburg 1996) may seem to be a non-mathematical, meaningless enquiry; for others who are in different phases of strategy change (see Lemaire and Siegler 1995 in Section 4.2) such self-referential discussions may prove to be effective.

### 5.2. Implications for Teaching and Learning

#### 5.2.1. Curriculum

Campione et al. (1988) juxtaposed three important areas of school learning: reading, writing, and mathematics. According to their analysis, fostering metacognitive components of these three basic skills was beyond the scope of curricula. The traditional mean of improving the traditional three Rs (Reading, wRiting, aRithmetic) was teaching more or less automatic skills, hence letting the child believe that skill automation is an aim, not a tool, and struggling learners receive heavier emphasis on drill-like skill practices. During the last three decades, decision-makers, scientists, and (to an increasing extent) in-service teachers do agree on the importance of fostering metacognition (Gourgey 1998). However, the methods of metacognition-based trainings might carry the danger of throwing the baby out with the bathwater, i.e., neglecting or marginalizing the massive amount of practice needed for smooth and automatic skill functioning. In line with our model depicted in Figure 1, and in line with recent empirical findings on the most important age range (7 to 11 years) for the development of brain fundamentals, our suggestion for curriculum developers is that metacognitive or strategic processes should not be labeled as target variables and should not appear in the timeline later than skill automation. Metacognitive processes are well needed *for* skill automation.

#### 5.2.2. Teaching and Learning

A most important curricular aim of elementary mathematics education is the smooth functioning of arithmetic skills, and arithmetic performance should be applicable in various contexts as embedded in word problems. In order to reach these curricular goals, didactical practices and principles have been handed down from generations to generations of teachers for several centuries. Of course, the role of the massive amount of time and practice in skill development is still unchallenged. What is new is the utilization of metacognition theory that has emerged over a couple of decades. Now that it is evident that metacognitive processes play their important role both in the learning process of new arithmetic strategies and in the application of arithmetic skills in word problem solving, teaching and learning should find the means by which teachers can support the development of their students.

Logan (1988) suggested that automaticity comes with ample practice in the same environment. From an educational point of view, this suggestion can be supplemented by recent developments in instructional methodology (including developments in educational tools and in teachers’ specialized content knowledge). Can we surpass or at least enrich the well-known practice-makes-perfect slogan? Kuhn (1995) suggested putting more emphasis on the top-down approaches in skill development. Borrowing the term from Karmiloff–Smith, “explicitation” may refer to learning processes in which the formerly implicit knowledge (whether being of declarative or procedural nature) becomes explicitly available to the mind. The study of means of such explicitation is still challenging (as we have seen in Section 4), but the practical methods would certainly involve instructional processes that have been extensively studied recently under the conceptual umbrella of metacognitive scaffolding. New educational tools like PhotoMath^1^ can help teachers to focus on learning targets rather than work with counting difficulties (Webel and Otten 2015). However, the importance of quick and flawless number fact retrieval remains an important curricular aim mainly due to the strong brain–mind correlates in this domain-specific construct.

Of course, teachers used metacognitive scaffolding even well before the advent of the term scaffolding. Csíkos’ (2016) results imply that teachers do implicitly teach addition strategies to 3rd-grade students. There were remarkable differences between students in their strategy use according to their affiliations with the schools, implying that two different mental calculation strategies were taught in the two participating schools. However, a transparent and efficient education system would require more widespread use of the best practices, especially if those practices have been empirically tested.

Among the educational implications stressed by Träff (2013), we highlight the importance of strengthening the retrieval of arithmetic facts. However, beyond what even lay people would suggest, i.e., mere drilling practice, he emphasized a kind of top-down direction which is embodied in using word problem solving and sharpening the approximate number abilities by means of the mental number line. The top-down approaches suggested by both Kuhn (2000) and Träff (2013) require teachers to possess specialized pedagogical content knowledge (Ball et al. 2008).

Teachers are (or should be) able to solve many different kinds of word problems almost automatically (while executing their classroom management duties), and some minutes later they can emulate their students’ task solving processes, illustrating their calculation processes by think-aloud sessions. The following description should be true for teachers: “in order to become competent at monitoring their own reflective reasoning, people must first acquire knowledge of the relevant reasoning norms, and only then are they able to notice shortcomings in their reasoning” (Fletcher and Carruthers 2012, p. 1369). For teachers’ professionalization and pondering towards the development of in-service and pre-service teacher training programs, Duffy (2005) emphasized the importance of “adaptive expertise”, a term that concisely describe what both students and teachers need in order to perform well in a given domain (Parsons 2012).

Teachers whose questions and classroom talk follow the principle of metacognitive scaffolding (Zimmerman 2007) use a kind of “cognitive processing language” (Hudson et al. 2018). Teachers using this kind of language support students’ strategy use in the long run, throughout the elementary school years. Thinking aloud while solving a task is certainly an effective way of metacognitive scaffolding already among preschoolers as revealed by Baten et al. (2017). To acquaint students with the importance of the ability to express their thought during math classes is also a powerful, reflective practice (Monaghan 2005) helping students get scaffolding. Of the factors that are necessary for metacognition-based collaboration and were studied by Goos and Galbraith (1996), mutual respect and the equal distribution of power may be fulfilled for an open classroom discourse.

Finally, we would like to take a more nuanced approach to Reusser’s (2000, pp. II/19) ideas, “As experienced teachers know: swotting up on number facts is fast and doable for everyone, however, hardly productive, while learning by understanding needs time and skillful scaffolding and mediation.” The clarification we propose is not labelling “swotting up on number facts” as only hardly productive, since it may be proven as a temporarily effective way of improving arithmetic performance. Furthermore, of course, both rote learning of number facts and acquiring arithmetic skills can be time-consuming, and their effectiveness depends on individual, task, and context variables as well. There are differences among the basic arithmetic operations in terms of the relative importance of number fact retrieval and calculation procedures (Peters and De Smedt 2018). The multiplication table (up to at least 10 × 10) is a common tool for strengthening students’ number fact retrieval, while teaching and practicing mental calculation strategies gets less emphasis. To the contrary, there use to be different mental subtraction strategies taught, while number fact retrieval takes a back seat in subtraction tasks.

As for the right understanding of what “teaching strategies” mean, Threlfall (1998, p. 6.) warns that teaching strategies should mean providing “lots of opportunities for children *to find their own way* [italicized by the author] through number challenges in an atmosphere of invention”. Because when strategy use is too automated, as a by-result, meaningless reliance on a given strategy may result in serious defect in development. As revealed by Allain et al. (2005), albeit persons with Huntington’s disease performed significantly weaker in a series of simple arithmetic word problems than control subjects did, on a task requiring the declaration that the task was unsolvable, no significant difference was found. It does not mean that Huntington’s disease may have some advantages regarding arithmetic strategy use, but it may indicate that regular classroom teaching can favor and over-automate the use of a privileged, albeit superficial task solving strategy.

Another alerting example comes from Nunes et al.’s (1993) book in which the discourse with a 12-year-old street vendor revealed how complex strategy he used when multiplying 35 by 10. As opposed to a strategy possibly taught in schools, i.e., multiplication by ten, it can be done simply by putting a zero to the right of the number. In that context, the child deliberately applied a series of steps involving additions, multiplications, and number facts to get the result. We would not claim that the street vendor’s strategy was inferior in any educationally relevant aspects; however, there is a clear distinction between the classroom context and the school of Life.

The main message of the current essay is: Do not take as granted that metacognitively rich learning and performance are necessarily good. However, do take as granted that metacognitively rich instruction, i.e., specialized content knowledge enriched by metacognitive scaffolding, seems to be the most promising way to support the development of arithmetic performance. More briefly: teach metacognitively but dispense metacognition in the spirit of “dosing as needed”.

## Data Availability

Not applicable.

## Note

1 Having written a simple addition like 5 + 2 onto a piece of paper, smart phones with PhotoMath application can immediately tell that the result is 7. Could it cause some kind of loss in terms of quick access to mental number facts?
